# The Role of Molar Mass in Achieving Isotropy and Inter-Layer Strength in Mat-Ex Printed Polylactic Acid

**DOI:** 10.3390/polym14142792

**Published:** 2022-07-08

**Authors:** Andrea Costanzo, Alice Poggi, Stan Looijmans, Deepak Venkatraman, Dan Sawyer, Ljiljana Puskar, Claire Mcllroy, Dario Cavallo

**Affiliations:** 1 Department of Chemistry and Industrial Chemistry, University of Genoa, 16146 Genova, Italy; andrea.costanzo@edu.unige.it (A.C.); alipoggi18.11@gmail.com (A.P.); 2 Department of Mechanical Engineering, Eindhoven University of Technology, P.O. Box 513, 5600 Eindhoven, The Netherlands; s.f.s.p.looijmans@tue.nl; 3 NatureWorks LLC, 17400 Medina Road, Suite 800, Plymouth, MN 55447, USA; deepak_venkatraman@natureworksllc.com (D.V.); dan_sawyer@natureworksllc.com (D.S.); 4 Helmholtz-Zentrum für Materialien und Energie GmbH, Albert-Einstein-Straße 15, D-12489 Berlin, Germany; ljiljana.puskar@helmholtz-berlin.de; 5 School of Mathematics & Physics, University of Lincoln, Lincoln LN4 7TS, UK

**Keywords:** material-extrusion, polylactic acid, weld strength, constitutive modelling

## Abstract

There has been extensive research in the field of material-extrusion (Mat-Ex) 3D printing to improve the inter-layer bonding process. Much research focusses on how various printing conditions may be detrimental to weld strength; many different feedstocks have been investigated along with various additives to improve strength. Surprisingly, there has been little attention directed toward how fundamental molecular properties of the feedstock, in particular the average molar mass of the polymer, may contribute to microstructure of the weld. Here we showed that weld strength increases with decreasing average molar mass, contrary to common observations in specimens processed in more traditional ways, e.g., by compression molding. Using a combination of synchrotron infra-red polarisation modulation microspectroscopy measurements and continuum modelling, we demonstrated how residual molecular anisotropy in the weld region leads to poor strength and how it can be eradicated by decreasing the relaxation time of the polymer. This is achieved more effectively by reducing the molar mass than by the usual approach of attempting to govern the temperature in this hard to control non-isothermal process. Thus, we propose that molar mass of the polymer feedstock should be considered as a key control parameter for achieving high weld strength in Mat-Ex.

## 1. Introduction

Additive manufacturing, more commonly known as 3-D printing, is a processing technology for different classes of materials that allows objects to be produced from models prepared with specific computer-aided design software [1,2,3,4]. Among the different techniques available, MatEx has a prominent role. In this technique, a filament of polymeric material is pushed through a nozzle, heated to significantly reduce its viscosity, and subsequently deposited layer by layer onto a build plate [5,6,7]. Thanks to a wide range of materials available, this technology is now used for prototyping in various sectors, including medical, engineering and automotive [8,9]. However, the use of this technology for practical applications is significantly affected by the anisotropy of the mechanical properties found in the printed products [10,11,12]. In particular, it has been found that the ultimate tensile strength is higher in the direction parallel to that of the filament deposition, while it strongly decreases in the perpendicular direction [13,14,15]. It is therefore clear that the final mechanical properties of the manufactured articles are strongly correlated to the quality of the bond that is established between the adjacent layers [16]. The fused filament fabrication (FFF) technique, in contrast to injection moulding, for instance, is unable to exert a strong pressure on the melt to obtain a homogeneous microstructure. In isothermal conditions, the strength of the interlayer bond is dependent on the welding time to the power one fourth, and the exponent is related to the diffusion process of the chains across the layer-layer interface [17,18]. However, the FFF deposition process is inherently non-isothermal, leading to the large temperature gradients in the welding area. As demonstrated by IR thermography measurements and by thermocouples, the deposition of successive layers leads to a periodic temperature fluctuation, ranging between a temperature close to that of the nozzle and at lower limit, to the temperature of the build plate [10,19,20,21]. The welding process is even more complicated in the case of semi-crystalline polymers, as it is strongly influenced by the crystallisation process [22]. The formation of the neck between the bonding filaments is only possible above the crystallisation temperature, due to a significant increase in the viscosity of the melt as the crystallisation temperature is approached [23]. Moreover, it is known that harsh printing conditions can largely influence the final mechanical properties. In previous works, it has been demonstrated that the use of low nozzle temperatures and higher printing speeds significantly reduce the quality of the weld [15,24,25]. By combining birefringence measurements and tensile tests with results from a continuum model, Costanzo et al. highlighted that the cause of the low weld strength for stronger processing conditions is the molecular orientation present at the interface between the layers [26]. Lower printing temperatures limit the time interval above the glass transition temperature and consequently the diffusion of the chains. At the same time, high deposition rates generate greater shear stresses on the polymer melt, favouring the alignment of the chains in the direction of the nozzle movement [27]. Polymer molecular features, in particular the average molar mass and its distribution, can also affect the final properties of the products. Recently, Levenhagen et al. showed that the addition of low molar mass polylactide to high molar mass polymer, resulted in a bimodal blend that drastically improves the adhesion between the layers [28]. They concluded that shorter molecules could diffuse faster at the interface, thus allowing for better welding and mechanical properties. Similarly, Srinivas et al. demonstrated for polylactic acid (PLA) that the interface stiffness itself can be improved by using an adequate enantiomeric composition and a multimodal molar mass distribution, including a fraction of low molecular weight species [29]. Along the same lines, in the study of poly-ether-ether-ketone (PEEK) printed samples, Xu et al. showed that a reduction of molecular mass can increase fluidity, reduce internal defects and, consequently, significantly improve the mechanical properties of the printed product [30]. Moreover, by employing a trouser tear test, Vaes et al. demonstrated that for nylon copolymers a decrease of the feedstock molar mass improved interlayer adhesion, due to the increased equivalent weld time [31]. However, the possible effect of residual orientation at the weld interface was neglected. To better understand the effect of varying molecular features on the weld properties of FFF parts, this paper proposes to examine the behaviour of different polylactide filaments, characterised by different molar masses and D-lactide co-monomer content, to find a correlation between the printing conditions, the final mechanical properties, and the intrinsic molecular characteristics of the polymers. We underline that the D-lactide content role in MatEx has not been explored yet, and therefore its effect on weld strength is presently unknown.

## 2. Materials and Methods

This study compared the behavior of six different polylactide filaments having different molar mass and D-lactide comonomer content. In general, the considered materials contain either low, intermediate, or high content of D-lactide comonomer. The approximate percentages are indicated, for the sake of simplicity, as 0, 4, and 12 mol%. Table 1 shows the list of materials used with the indication of their molecular characteristics, including the average number of entanglements per chain (the molar mass of a chain section between entanglements is *M_e_* = 9 kg mol^−1^ [26].

All materials were characterised by differential scanning calorimetry analysis (DSC) using a DSC 250 from TA Instruments (New Castle, DE, USA). The samples were first molten at 210 °C to eliminate the previous thermal history, and subsequently subjected to a cooling/heating cycle at 10 °C/min to study their intrinsic characteristics. Rheological analysis was performed using a HR 10 rheometer from TA Instruments (New Castle, DE, USA). The materials were prepared in the form of plates with a compression molding press and subsequently subjected to frequency sweep tests at different temperatures, consistent with the respective recommended processing ranges. Specifically, the rheological measurements were carried out in a range of angular frequencies from 0.1 to 100 rad/s, with an amplitude strain of 2% in the linear viscoelastic regime, repeating the measurement at six different temperatures for each material. The rheological measurements for the PLA_0_94k material were performed at a lower temperature range with respect to the others due to its incipient crystallisation at lower temperatures. The time-temperature superposition analysis to obtain the master curves and calculate the rheological shift factors was carried out using the TRIOS software from TA instruments. The specimens for weld strength measurements were printed using an Intamsys Funmat HT 3D printer (Shanghai, China), equipped with a 0.4 mm diameter nozzle. The chosen geometry is a free-standing open cube (dimensions 4 cm × 4 cm × 4 cm) consisting of single layers of material superimposed on each other, with a layer thickness of 0.2 mm, the same as used in previous works [15,26]. The sample with the chosen geometry was designed with the Tinkercad software and subsequently converted into G-code format with the Ultimaker Cura slicing software [32]. Table 2 summarises the explored printing conditions for the materials. Since structurally sound specimens could not be achieved for low molar mass materials at low deposition rates, the print speed ranges explored were 20–120 mm/s and 60–120 mm/s for higher molar mass and lower molar mass materials, respectively.

The samples were subjected to a tensile test to determine the strength of the weld as a function of the printing conditions [15,33]. Each printed cube was cut out along the lateral walls, and from each wall a rectangular sample was subsequently obtained using a pneumatic punch. Specifically, the samples used for the mechanical tests had the following dimensions: 40 mm in height, 13 mm in width, and about 0.45 mm in thickness. The direction of deposition of the layer was oriented at an angle of 90° with respect to the direction of tensile deformation. Tensile tests were performed using an Instron 5565 S/NO H1505, with an initial distance between the clamps of 12 mm and a separation rate (tensile speed) of 6 mm/min. The thickness of the adhesion surface between the adjacent layers, required to correctly evaluate the stress experienced by the sample, was measured using a stereoscope. Figures of the adopted geometry for mechanical testing are provided in previous works [26,27].

To measure the molecular orientation in the printed specimens, we made use of modulated polarisation spectroscopy. The experiments were conducted at the IRIS Infrared beamline of the Helmholtz-Zentrum Berlin synchrotron facility BESSY II. The setup is composed of a Nicolet Nexus 870 FTIR spectrometer coupled to a Nicolet Continuum infrared microscope and a Hinds PEM-90 II photoelastic modulator (PEM) unit to generate the orthogonally polarised light. The PEM consists of a ZnSe crystal which, when stretched and compressed, creates birefringence of infrared light in the material, inducing a periodic rotation of the polarisation plane from 45° to 0° and 90° at a high frequency. In this way, the doubly modulated IR beam containing alternating polarisation is focused on the sample and collected at the dual channel detector of the microscope. The signals of the two orthogonal polarisation states are split from the modulated interferogram by a demodulator generating the difference (II − ⊥) and sum (II + ⊥) interferograms. With the Fourier transform of these two interferograms, one obtains the normalised differential absorbance spectrum, or so-called polarisation modulated dichroic difference (PMDD), defined by Equation (1):

(1) $$I_{PMDD}=\frac{I_{\mathrm{II}}-I_{⊥}}{I_{\mathrm{II}}+I_{⊥}}$$

where *I_II_* is the intensity at the detector when the incident light is polarised parallel to the reference axis of the sample, and *I*_⊥_ the intensity at the detector when the light is polarised perpendicular with respect to the sample. For more details about this set-up, readers can refer to [34,35]. By changing the two orthogonal polarisation states and obtaining the differential absorbance spectra, it is possible to determine the local orientation of dipoles, and therefore the local chain conformation. All measurements were performed in transmission mode using two IR reflective objectives (Schwarzschild, 0.65 N.A.) and a MIR liquid nitrogen cooled HgCdTe detector. Spectra were taken in the range from 650 cm^−1^ to 4000 cm^−1^ with an aperture size of 50 × 50 μm and 6 cm^−1^ spectral resolution. Each point was accumulation of 256 scans. The intrinsic sample reference axis was chosen to be the printing direction. The specimens on which polarisation modulation spectroscopy measurements were carried out were printed with a rectangular shape and dimensions 1.5 cm × 1 cm × 0.5 cm, maintaining the same deposition direction for each layer. Part of these samples were cut to a size which could fit the sample holder of a manual microtome (Leica RM2235), and then cut at room temperature in the direction parallel to that of printing. The final thickness of the obtained slices was 5 μm, an optimal size for avoiding saturation of the PLA absorption bands. Using the Omnic software, a rectangular map of 80 points was selected on each sample. The map consisted of 4 lines containing 20 points each with 20 μm step size between the points.

The analysed sample slices were large enough to allow the observation of at least one interface between different printed layers and thus have information on the possible distribution of the orientation.

## 3. Results

### 3.1. Materials Characterisation

**Figure 1:**
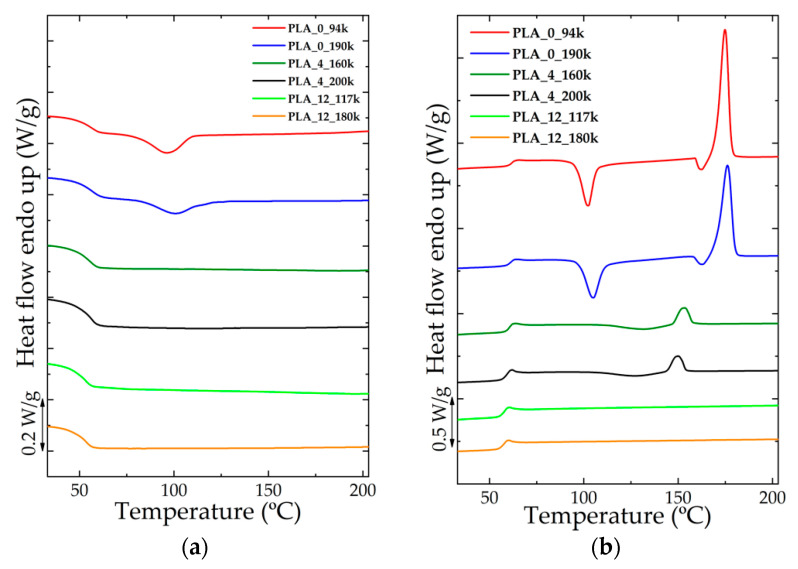
Cooling ramp (**a**) and heating ramp (**b**) obtained by DSC calorimetric analysis for the six materials.

DSC cooling and heating ramps at 10 °C/min for all the investigated materials are reported in Figure 1. As demonstrated by the DSC calorimetric analysis, these materials exhibited a different behavior as the percentage of D-lactide comonomer varied. A higher percentage of monomer, in fact, strongly limited the possibility of the materials to crystallise, making their structure totally amorphous upon standard cooling condition. Conversely, the absence of D-lactide monomer units in the polylactic acid chains allowed obtaining a semi-crystalline structure. The final degree of crystallinity thus decreased with increasing comonomer amount. On the other hand, at the same comonomer level, a variation of the molar mass did not meaningfully affect the thermal behavior of the material.

**Figure 2:**
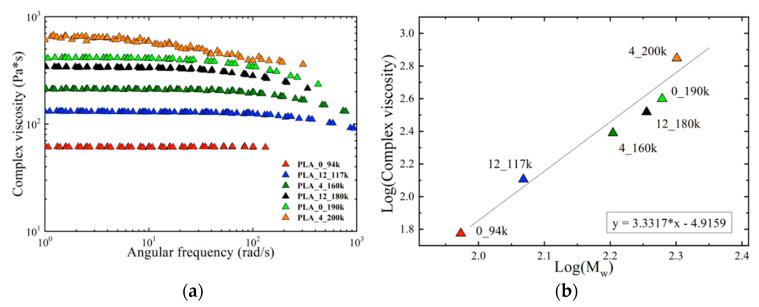
(**a**) Complex viscosity obtained with temperature–time superposition analysis at the reference temperature of 190 °C as a function of the angular frequency for all materials; (**b**) logarithm of the Newtonian viscosity as a function of the logarithm of molar mass for all the materials. The angular coefficient of the trend line confirms that the molar mass is the main parameter that influences the viscosity.

Complex viscosity curves obtained with temperature–time superposition analysis at the reference temperature of 190 °C for all materials are reported in Figure 2a.

It can be observed that for all materials the complex viscosity initially remained constant as the angular frequency varied (Newtonian plateau) up to a certain value, after which the viscosity begins to decreasd. As expected, viscosity increased with molecular mass, while the D-lactide content did not seem to influence its behavior. This can be verified when the logarithm of the Newtonian viscosity is plotted as function of the logarithm of molar mass. As can be seen in Figure 2b, the data approximately followed one straight line with a slope close to 3.4, confirming that molar mass is the relevant variable which governs the viscosity, in agreement with previous literature [36,37,38]. In light of this, and of the negligible degree of crystallinity present in the printed PLA copolymers, we believe that molar mass will have a more marked effect on the welding process, rather than D-lactide content.

### 3.2. Mechanical Testing

**Figure 3:**
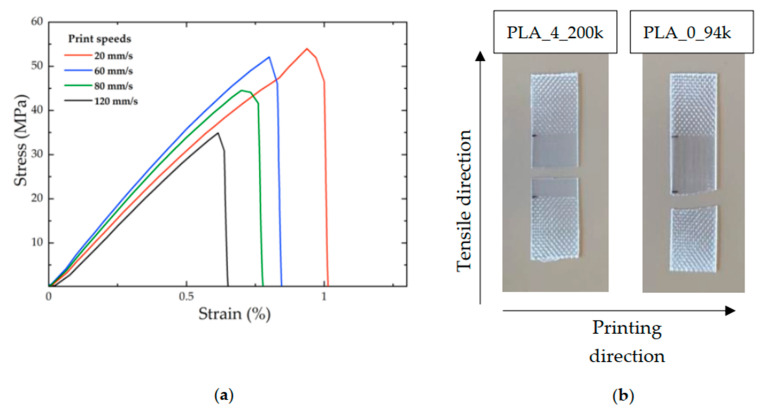
(**a**) Examples of stress/strain curves for PLA_4_200k samples printed at 200 °C at four different print speeds (20, 60, 80, and 120 mm/s) and for the sample obtained by compression molding; (**b**) examples of fractured samples following mechanical tensile testing for PLA_4_200k and PLA_0_94k printed at a nozzle temperature of 210 °C and print speeds of 20 mm/s and 60 mm/s, respectively.

Figure 3a shows an example of stress/strain curves for PLA_4_200k samples printed with a nozzle temperature of 200 °C and at four different print speeds. The stress to which the samples were subjected was calculated by dividing the area of the weld region previously measured for each sample by using a stereoscope. It is evident that, for the chosen nozzle temperature, the elongation at break and the stress at break decreased with increasing printing speed. Qualitatively, the type of fracture of the specimens was different at the various printing speeds. In particular, at low print speed the failure occurred “predominantly” bulk-like rather than along the weld. The opposite was true at high print speed. Figure 3b shows two examples of broken samples after mechanical testing, related to PLA_4_200k and PLA_0_94k both printed at 210 °C and at similar rates (20 mm/s and 60 mm/s, respectively). In the case of the high-molar-mass polylactide, the breaking occurred completely along the weld line, so the tensile test can be considered as representative of the weld strength. In the second case, for low-molar-mass polylactide, the fracture line seems to resemble a bulk-type breaking, spanning more than one weld line, and beginning at the clamps. These pictures are representative of the prevailing failure mechanism of high- and low-molar-mass samples, respectively.

**Figure 4:**
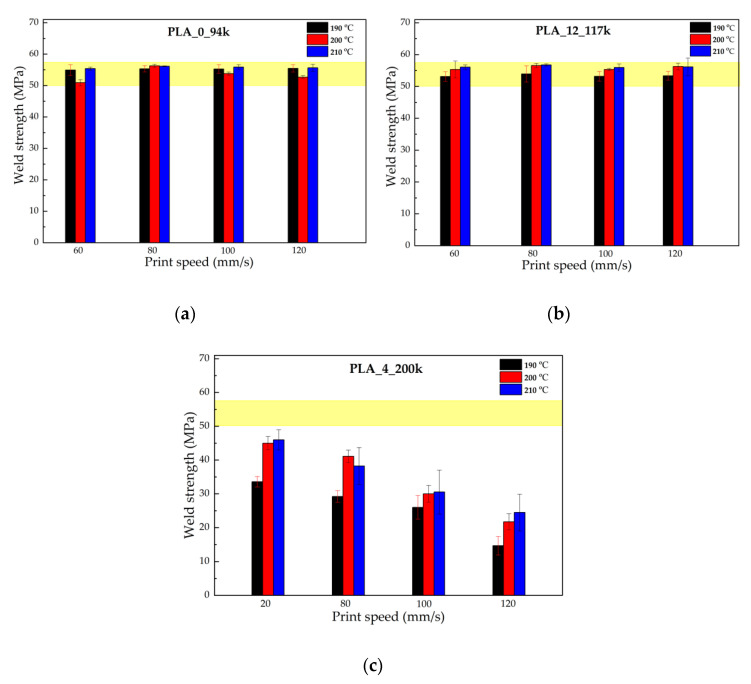
Weld strengths as a function of printing conditions for (**a**) PLA_0_94k, (**b**) PLA_12_117k, and (**c**) PLA_4_200k. The shaded area in each graph corresponds to the range of bulk strength values measured using dog-bone-shaped specimens obtained by compression molding.

Figure 4 summarises the weld strengths, i.e., the maximum stress in the stress–strain curves, for PLA_0_94k, PLA_12_117k and PLA_4_200k measured at different printing conditions. The shaded areas in each graph correspond to the range of bulk strength values measured using dog-bone-shaped specimens obtained by compression molding.

**Figure A1:**
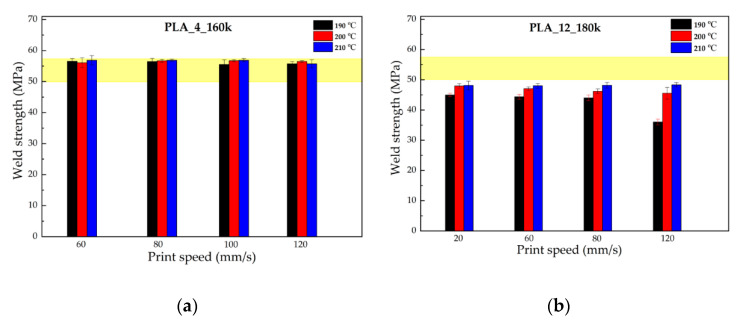

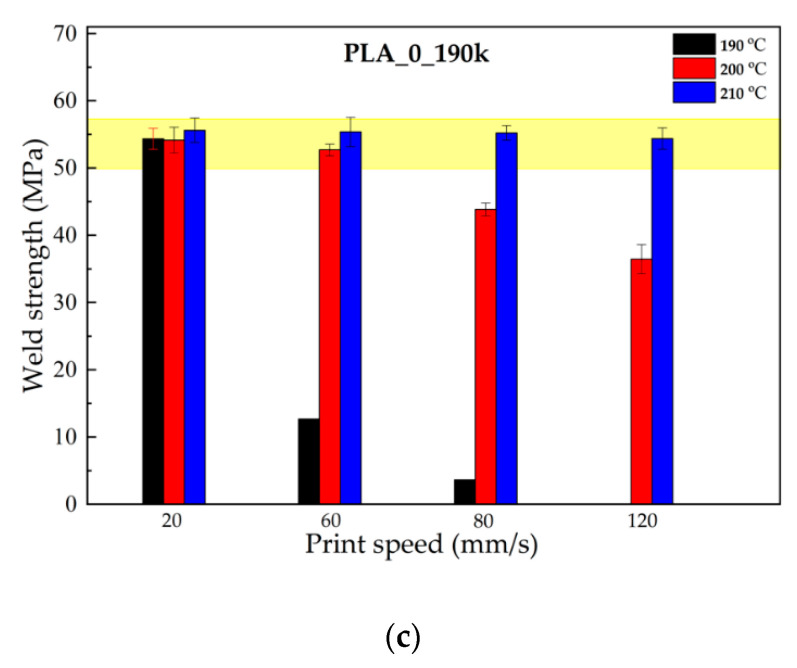
Weld strength as a function of different printing conditions for (**a**) PLA_4_160k, (**b**) PLA_12_180k and (**c**) PLA_0_190k.

Observing the plots (comparing Figure 4a with Figure 4b), it is easy to see that the percentage of D-lactide monomer has little influence on the final mechanical properties. The weld strength is instead closely related to the molecular mass of the material. In fact, the weld strength of the low molecular weight materials does not seem to be influenced by the processing conditions; in the case of PLA_0_94k, the weld strength values were approximately constant with extrusion temperature and print speed. This contrasts to the sample of highest molecular weight, PLA_4_200k, for which there was a distinct decrease in mechanical properties with stronger printing parameters (lower nozzle temperatures and higher deposition rates); it can be seen that increasing the nozzle temperature improved the adhesion strength. The same data for the other three materials are reported in Appendix A (Figure A1).

## 4. Discussion

Since the bulk strength of a polymer is known to typically increase with molar mass [39,40], and in general we observe that low molecular weight samples achieve a greater tensile strength, this suggests that the printing process is affecting the underlying microstructure within the weld region. We propose that the decrease in mechanical properties (weld strength) we observed is caused by residual chain orientation at the interface. Indeed, residual orientation at the glass transition is expected to increase with molar mass and print speed and to decrease with increasing nozzle temperature. To test our hypothesis, in the next section we measure the anisotropy in the weld region using polarisation-modulated infrared microspectroscopy. These results are further supported by model predictions of residual alignment in the weld, which we then compare directly to our weld strength measurements.

### 4.1. Measuring Weld Anisotropy

To confirm that the decay of the tensile stress as a function of print speed for the high-molar-mass sample is due to a residual orientation of polymer chains at the interface between the layers, polarisation modulated infrared microspectroscopy was used. These measurements were carried out on two materials possessing the highest and the lowest molar mass, namely PLA_4_200k and PLA_0_94k respectively.

**Figure 5:**
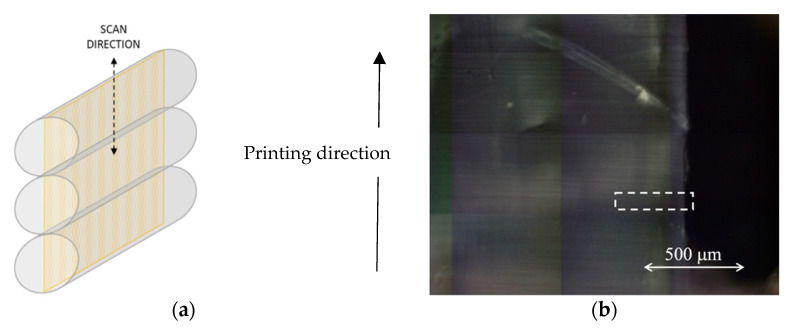
(**a**) Sketch of the microtoming operation that was adopted for preparing the samples for the PM-IR measurements; (**b**) an example of a PLA_4_200k sample printed at 20 mm/s and 210 °C to be scanned with PM-IR. The X-Y axis represents relative distance in microns on the microscope sample stage.

Figure 5a shows a representation of the microtoming cut made on the two printed samples. The samples were microtomised in a direction parallel to that of printing, thus obtaining slices of filaments with a thickness of 5 μm. It should be underlined that the manual microtoming procedure, with the difficulties of handling the small samples, did not allow cutting the printed objects exactly through the center of each layer. As such some uncertainty in the vertical position of the slice within the layer exists, we were forced to take measurements from three adjacent slices per sample to obtain representative results. Figure 5b shows an example of visible light image of a microtomed section from PLA_4_200k printed at 20 mm/s and 210 °C seen under the confocal IR microscope before the PM-IR scan.

**Figure 6:**
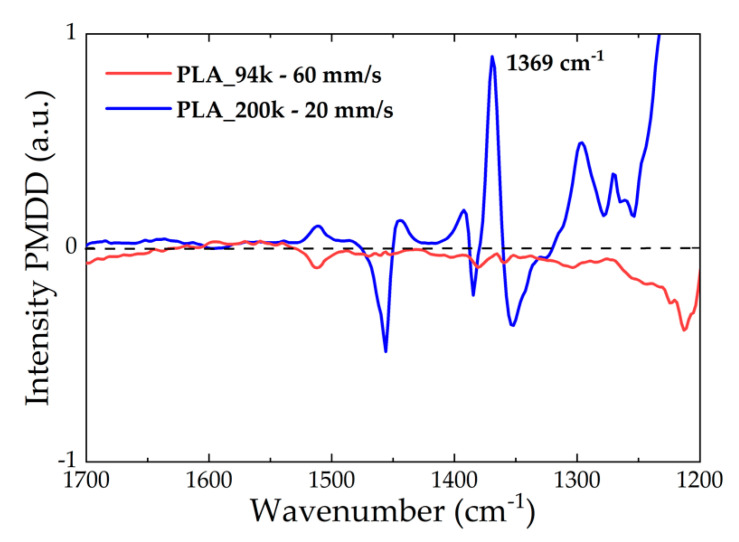
Comparison of the PMDD spectra acquired for PLA_4_200k printed at 20 mm/s and PLA_0_94k printed at 60 mm/s.

Figure 6 shows an example of two polarisation modulation dichroic difference spectra measured for a sample of PLA_4_200k and one of PLA_0_94k printed at 210 °C using a speed of 20 mm/s and 60 mm/s, respectively. It is evident that the PMDD differential absorption spectra were very different for the two materials. For the low-molecular-weight material, the PMDD intensity remained close to zero for all the measured range of wavenumbers. In contrast, the spectrum of PLA_4_200k showed more anisotropic absorbance as evident by stronger intensity positive and negative bands at precise wavenumbers, which indicated the presence of molecular anisotropy. The peak appearing at approximately 1383 cm^−1^ which corresponds to the symmetric deformation of the CH_3_ group [41] was chosen as a reference band to evaluate the PMDD intensity in different regions, due to its sensitivity to molecular orientation.

**Figure 7:**
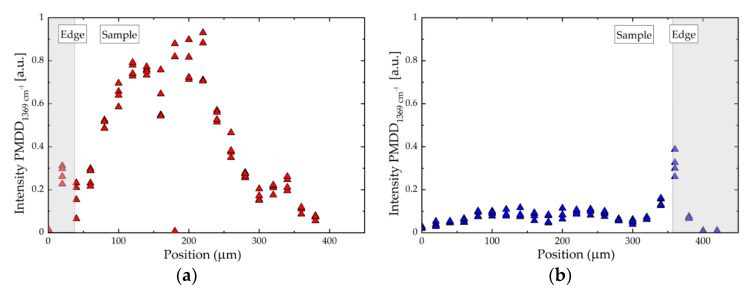
PMMD intensity for (**a**) PLA_4_200k and (**b**) PLA_0_94k as a function of position in the sample. Dark areas highlighted on the plots correspond to the air–sample interface.

Figure 7 reports the PMDD intensities calculated for the two materials as a function of the position in the sample. In both cases, the acquisition of the spectra started from outside the sample, thus presenting a clear separation surface between the air and the material itself. For the case of PLA_4_200k (Figure 7a) it can be noted that, moving towards the central region of the sample, there was a net increase in intensity, which remained at high levels for a length of about 200 µm, and then fell again. This behaviour, on the other hand, did not occur in the case of low molar mass PLA, for which, throughout the thickness analysed, there was no meaningful variation in intensity (Figure 7b).

The results of these measurements allow us to highlight the different degree of molecular anisotropy that develops in the two materials during the printing process and is frozen in at solidification. For the lower-molar-mass PLA_0_94k sample, no residual orientation was measured after the printing process, while for PLA_4_200k a measurable and spatially dependent degree of anisotropy was observed. We expect that this highly oriented region corresponds to the interface between the different layers (as predicted by the model); however, due to the imprecision in the microtoming process, this is difficult to confirm.

### 4.2. Modelling Weld Anisotropy

To support our hypothesis, molecularly aware modelling of the FFF processing of our selected materials subject to the chosen printing conditions was carried out; here we show results for fixed print speed 120 mm/s and the temperature range 190–210 °C. We refer the reader to our previous works for full details of the model, which has been applied to various materials [26,42,43].

In summary, we modelled flow through the heated nozzle followed by deposition of a single cylindrical filament with an appropriate constitutive equation (Rolie-Poly) that captures the dynamics of a linear monodisperse polymer melt. This flow deforms the microstructure so that the polymers become stretched and aligned in the direction of deposition; we assume that this alignment reduces the entanglement of the polymer network. As the flow is dominated by shear in the nozzle, the model predicts higher degrees of deposition near the surface of the deposited filament.

Upon deposition, the filament was cooled by employing the heat equation with an axisymmetric boundary condition. This boundary condition was determined from experimental measurements of a typical cooling profile according to the material and printing conditions. Here the velocity gradients cease, the flow-induced deformation begins to relax, and entanglements reform according to the constitutive model. We infer that the polymers will begin to diffuse at a layer-layer interface via reptation. Once the glass transition temperature is reached, this diffusion/relaxation/re-entanglement process is arrested. This was captured in the model via a simple Williams–Landel–Ferry (WLF) dependence of the polymer relaxation times (Equation (2)), as in previous work [26,27],

(2) $$a\left(T\right)=exp\left(\frac{C_{1}\left(T-T_{0}\right)}{C_{2}+T-T_{0}}\right),$$

where the shift factors *C_1_* and *C_2_* are obtained from time–temperature superposition as described above. At this point, we probed the state of the weld region (i.e., the surface of the deposited filament) via three molecular features: the degree of alignment, the degree of entanglement, and the degree of interdiffusion (not reported here). We found that the degree of interdiffusion always exceeded the polymers’ radius of gyration, as seen in previous work [26], for all the values of *Z_eq_*.

**Figure 8:**
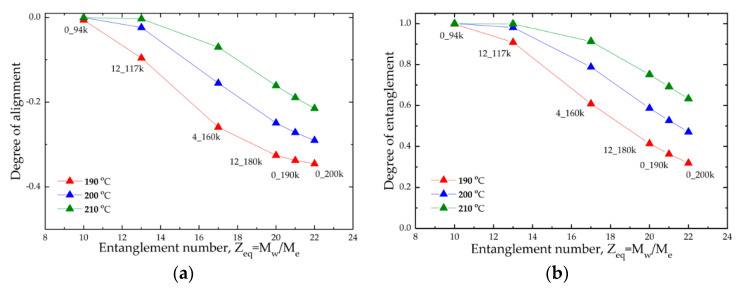
(**a**) Degree of alignment and (**b**) degree of entanglement calculated for different nozzle temperatures as a function of *Z_eq_* = *M_w_*/*M_e_*.

The degree of alignment and the degree of entanglement are plotted in Figure 8 for our samples of varying molecular weight for print speed 120 mm/s and a range of nozzle temperatures. The molecular weight of the material corresponds to an equilibrium entanglement number of the melt *Z_eq_* = *M_w_*/*M_e_*, where *M_e_* is the molecular weight of a single section between entanglements, which is obtained from fitting the rheological master curve to the Likhtman–McLeish theory via RepTate software [44] as in previous work [27,28]. We found that the degree of alignment and therefore the degree of disentanglement increased strongly with increasing molecular weight. This is a direct consequence of polymer reptation time scaling with the entanglement number via Equation (3)

(3) $$\tau_{d}\propto Z_{\mathrm{eq}}^{3}$$

That is, higher molecular weight samples (e.g., *Z_eq_* = 22) have a significantly longer reptation time meaning that not only do they deform more during the deposition flow, but the time to relax back to an isotropic state is much longer. Consequently, a greater degree of molecular anisotropy is trapped in at solidification. This is in contrast to low molecular samples (e.g., *Z_eq_* = 10), which have sufficient time to fully relax before the onset of the glass transition so that the weld region is isotropic on solidification.

The print temperature has the biggest influence on this behaviour. As shown in Figure 8, the degree of molecular anisotropy in the weld region can be significantly reduced by increasing the print temperature. This is due to the temperature dependence of the reptation time (Equation (4)):

(4) $$\tau_{d}\propto a\left(T\right),$$

which diverges exponentially near to the glass transition temperature according to the WLF equation. As the reptation time is not affected by shear, print speed has only a small effect on these results. 

By comparing Equations (1) and (2), it is clearly more effective to control the polymer reptation time, and therefore anisotropy in the weld, by adjusting the entanglement number of the melt via the molecular weight opposed to managing the temperature profile during this hard-to-control non-isothermal process.

### 4.3. Modelling vs Experiments

**Figure 9:**
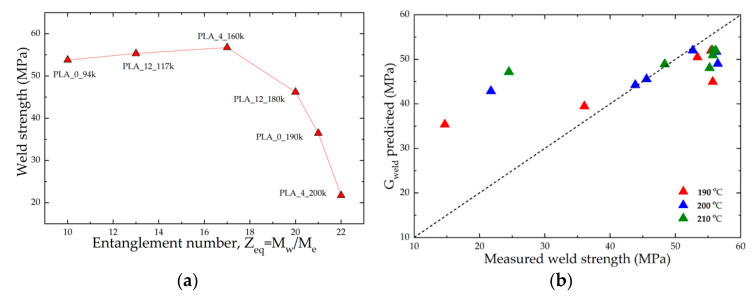
(**a**) Weld strength measured for samples printed at 200 °C and 120 mm/s as a function of *Z_eq_* = *M_w_*/*M_e_*; (**b**) Weld strength, *G_weld_*, predicted by the model (see Equation (5)) as a function of measured weld strength for all the explored nozzle temperatures and print speeds.

Figure 9a reports the weld strength for samples measured at 200 °C and 120 mm/s as a function of the entanglement number *Z_eq_* = *M_w_*/*M_e_* for all the investigated materials. The observed trend confirmed that high molar mass PLAs are characterised by a lower weld strength than lower masses. Moreover, the trend is in line with the degree of entanglement calculated by the model and displayed in Figure 8b. It is therefore possible to attribute the decrease of weld strength for higher molar mass to the presence of residual chain alignment at the weld region [26,27].

The PM-IR molecular orientation measurements in the two materials shown in Figure 7 corroborate the modelling results reported in Figure 8a. In fact, the material with the highest molar mass and entanglement number showed the larger degree of chain alignment in the printing direction, while the residual alignment was negligible for the low molar mass sample, due to the intrinsically faster dynamic which allows the relaxation of any orientation before cooling below the glass transition temperature. Therefore, the counter-intuitive molar mass dependence on molecular weight shown in Figure 9a is captured by the molecularly aware model, and is attributed to residual molecular alignment, as confirmed by PM-IR measurement on selected samples. A comparison between the measured and predicted values of weld strengths is shown in Figure 9b. By assuming a fracture mechanism based on crazing [28], the values of weld strength *G_weld_* are calculated according to Equation (5):

(5) $$G_{\mathrm{weld}}\propto {\left(1-\frac{p}{q}\frac{1}{\bar{v}Z_{eq}}\right)}^{2}$$

where *p* is the polydispersity index, *q* is a measure of the fraction of strands that survive fibrillation during a tear and is found to be *q* ≈ 0.6 in [45] and $\bar{v}$ is the final degree of entanglement at the weld. Figure 9b shows that there is good agreement between the model predictions and the experiments for the middle range of weld strength corresponding to the middle range of entanglement numbers. However, there is some discrepancy at the extremes.

There are a number of reasons for this deviation.

1. The High-Strength Regime. The model clearly underestimates the high strength regime, corresponding to the lowest molecular weight samples. This can be attributed to the temperature profiles employed by the model. Temperature measurements were carried out on the first deposited layer, which serve as an axisymmetric boundary condition in the model and provide the most extreme cooling profile experienced during the build. However, breaking occurs always at layers deposited at intermediate heights in the sample, where the cooling rate is less affected by the build plate [15,29]
2. The Low-Strength Regime. On the other hand, the model overestimates the weld strength in the low-strength regime corresponding to the high-molecular-weight samples. This may be indicative of the unsuitability of the crazing model to properly describe the failure of PLA welds with frozen-in orientation. Another possible reason is the existence of a low degree of crystallinity at the weld, which could effectively decrease the strength of the inter-layer bonding [31,46].

Despite this, the qualitative and semiquantitative agreement between Figure 8a and Figure 9b suggests that the physics behind the phenomenon were correctly captured, that is, the degree of residual alignment in the weld region is governed by the time taken for the flow-induced deformation to relax before the onset of the glass transition; thus, the dominant factors are the time above the glass transition temperature and the relaxation time of the polymer. The model (Figure 8) demonstrated that it is more effective to shorten the polymer relaxation time by reducing the molecular weight, compared with increasing the time above the glass transition temperature by attempting to control the thermal history. This result agrees with experiments (Figure 9).

In particular, for the PLA samples studied in this work, simply reducing the average molecular weight of a typical feedstock from 200 kDa (*Z_eq_* = 22) to 160 kDa (*Z_eq_* = 17) ensures that bulk strength is achieved in the weld for all print conditions. Increasing the nozzle temperature is not as effective (see Figure 4); the weld strength of a typical 200 kDa feedstock remains significantly less than the bulk strength at the highest nozzle temperature of 210 °C, particularly for high print speeds.

## 5. Conclusions

We investigated the interlayer strength achieved from a range of poly-lactic feedstock ranging in D-lactide content and average molar mass. Mechanical testing confirmed that molar mass has a strong effect, with strength decreasing significantly with entanglement number, whereas the effect of the D-lactide content is small. These measurements contrast expectations that increasing the number of entanglements increases the strength of a polymer, as evident in samples processed by more traditional methods, e.g., compression molding. Since, geometrically, all samples were similar, we conclude that the printing process alters the microstructure of the weld.

We used a combination of experimental polarisation modulated infra-red microspectroscopy measurements and continuum modelling to show that a significant degree of molecular anisotropy is frozen into the sample upon solidification for high-molar-mass samples (Figure 7a), whereas low-molar-mass samples exhibited isotropy (Figure 7b). The model suggests that this residual anisotropy is localised near to the surface of a deposited filament, as seen in previous work [26], resulting in a partially entangled weld region that reduces interlayer strength. This prediction is in qualitative agreement with the experimental measurements of the weld strength.

Achieving molecular isotropy in the weld is clearly an interplay between molecular weight and temperature. However, whilst print temperature is commonly implemented as a control parameter, varying the molecular weight of the feedstock is often overlooked and may be more effective, as demonstrated in this work. Thus, we propose that molecular weight should be considered as a key control parameter in the MatEx process; provided that *Z_eq_* > 6, lowering the molecular weight of the feedstock will achieve stronger builds due to a more isotropic weld.

## Figures and Tables

**Table 1:** Materials.

Adopted Name	Molar Mass *M_w_* (g/mol)	Approx. D-Lactide Content (mol %)	*Z_eq_* = *M_w_*/*M_e_*
PLA_0_94k	94.000	0	10
PLA_0_190k	190.000	0	21
PLA_4_160k	160.000	4	17
PLA_4_200k	200.00	4	22
PLA_12_117k	117.000	12	13
PLA_12_180k	180.000	12	20

**Table 2:** Printing parameters.

Nozzle temperature	190–210 °C
Print speed	20–120 mm/s
Build plate temperature	40 °C
Chamber temperature	30 °C

## Data Availability

The data presented in this study are available on request from the corresponding author.
